# Generalized Granuloma Annulare Associated With Dupilumab Therapy

**DOI:** 10.7759/cureus.27439

**Published:** 2022-07-29

**Authors:** Kendall Phelps-Polirer, Bailey L Alkhatib, Charles Davis

**Affiliations:** 1 Internal Medicine, USF Health, Bradenton, USA; 2 Infectious Disease, University of South Carolina School of Medicine, Columbia, USA; 3 Dermatology, William Jennings Bryan Dorn Veterans Affairs Medical Center, Columbia, USA

**Keywords:** granulomatous drug reaction, granuloma annulare, atopic eczema, dupilumab, atopic dermatits

## Abstract

Atopic dermatitis is a condition characterized by xerotic and pruritic skin. While the onset of the disease is usually in childhood, it may persist into adulthood. First-line treatments include adequate moisturization, avoidance of irritants, and the application of topical corticosteroids. Dupilumab is a biologic therapy, approved for moderate-to-severe atopic dermatitis, that dampens the pruritus sensation by inhibiting the downstream effects of the T helper cell type 2 pathway by binding to the interleukin-4 receptor α subunit. The monoclonal antibody is typically well-tolerated.

We present a novel case of the development of generalized granuloma annulare after treatment with dupilumab. A 71-year-old male with a history of hypertension, hyperlipidemia, chronic kidney disease, gout, and bipolar disorder presented to clinic with biopsy-proven severe atopic dermatitis. First-line treatments had been exhausted, and the patient was not an ideal candidate for traditional systemic options secondary to his poor renal function. Therapy with dupilumab was initiated and continued for two years until the patient developed biopsy-proven generalized granuloma annulare. At this time, dupilumab was discontinued and the pharmaceutical company was made aware of this side effect.

## Introduction

Atopic dermatitis (AD) is a prevalent and chronic inflammatory disease of the skin that has recently been the focus of various epidemiological, basic science, and clinic research endeavors [1]. AD is characterized by pruritus and sleep disturbance and affects 10% of adults in the United States [2]. Pruritus is a hallmark of the condition that is responsible for much of the disease burden [1]. The interplay between one's environment and genetic and immunologic composition contributes to an undermined skin barrier and dysregulation of the immune system [1]. The cutaneous inflammation is driven by T helper type 2 (Th2) mediated cytokines [3]. This increased understanding of the pathogenesis of atopic dermatitis has potentiated the discovery of novel therapeutic targets [3]. 

Dupilumab is a new biologic therapy that specifically targets a distinct immune pathway and its cytokines and receptor [3]. Dupilumab is a human monoclonal antibody that binds to the interleukin-4 (IL-4) receptor α subunit and inhibits the action of both IL-4 and interleukin-13 (IL-13), the interleukins involved in mediating the itch sensation [4]. It is currently the only biologic medication approved by the United States Food and Drug Administration for anyone six years and older with moderate to severe AD whose condition is not adequately controlled with topical prescription therapies or if other therapies are contraindicated [4]. Studies have shown that dupilumab is well-tolerated and results in significant improvement in AD symptoms. Reported side effects of dupilumab include injection site reactions, conjunctivitis, blepharitis, oral herpes, keratitis, eye pruritus, other herpes simplex virus infection, and dry eye [4].

We present a case report of a 71-year-old male with severe atopic dermatitis who developed generalized granuloma annulare (GGA) after treatment with dupilumab. To the best of our knowledge, this is the first case of granulomatous dermatitis associated with the use of dupilumab. 

## Case presentation

A 71-year-old African-American male with a past medical history of hypertension, hyperlipidemia, chronic kidney disease, gout, and bipolar disorder presented to the clinic with biopsy-proven severe atopic dermatitis since childhood. Current medications included amlodipine, simvastatin, aripiprazole, cholecalciferol, hydroxyzine, and omeprazole. The patient had previously been prescribed allopurinol, but he discontinued this medication four months before his initial presentation and two and a half years prior to the initiation of dupilumab. 

He reports his AD had flared three times in the last year despite treatment with class I topical steroids, narrow-band ultraviolet B therapy, steroid injections, and mycophenolate mofetil. Further treatment options for AD refractory to conventional therapies were explored. Systemic medications used in the treatment of AD such as cyclosporine, azathioprine, and methotrexate were considered, but due to the patient’s poor renal function the use of these drugs was contraindicated. Due to the refractory AD, treatment with biologic therapy was indicated, and he was started on dupilumab injections every two weeks. After 10 weeks of treatment, the patient reported significant improvement in his AD symptoms. After three months of treatment, he achieved clear skin and physician global assessment score is 1.

**Figure 1:**
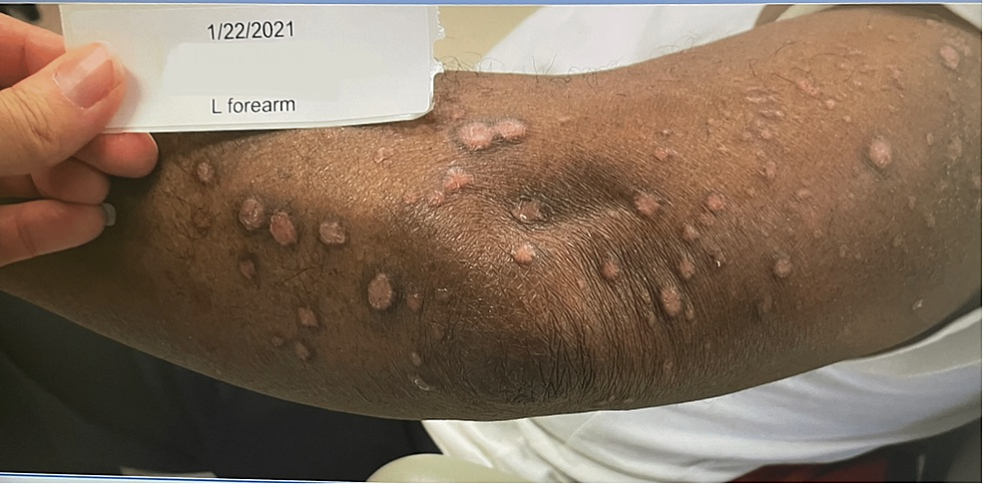
Characteristic annular erythematous papules and plaques over the patient's left upper lateral arm and forearm

After two years of treatment with dupilumab, the patient developed multiple annular well-defined skin-colored plaques on the forehead, back, and bilateral upper and lower extremities as seen in Figure 1. He stated the lesions were somewhat pruritic, but he was otherwise asymptomatic. A tangential skin biopsy of the patient’s left forearm was performed and demonstrated multinucleated histiocytes palisading around altered collagen with rare eosinophils present and occasional neutrophilic infiltrate in the areas of altered collagen. Staining revealed mucin within the altered collagen and interstitially in the dermis. The histopathologic findings of altered collagen and dermal mucin were consistent with granulomatous dermatitis, with generalized granuloma annulare favored. The differential diagnosis included interstitial granulomatous drug reaction (IGDR), but the lack of improvement four weeks after discontinuing dupilumab made this less likely.

Dupilumab was discontinued and the patient was placed on an eight-week trial of doxycycline 100 mg twice a day for its anti-inflammatory effects and clobetasol ointment twice a day for the inflamed GGA areas. No improvement was seen with doxycycline. The patient was seen at a follow-up visit four weeks after discontinuing dupilumab with no improvement of his skin. He was told to restart triamcinolone 0.1% ointment and phototherapy. He was advised that GGA would likely resolve on its own, and treatment would be targeted toward decreasing his symptoms.

## Discussion

Granuloma annulare (GA) is a noninfectious, self-limiting cutaneous inflammatory disorder. Patients are asymptomatic and classically present with papules in an annular configuration ranging in hue from skin-colored to erythematous [5]. Histopathologically, GA is characterized by necrobiotic collagen associated with an infiltration of histiocytes and lymphocytes [5]. In addition to the ubiquitous finding of collagen degeneration, dermal mucin was evident in 24 presentations, or 80% of cases [6]. The determination of an assured diagnosis of GA is based on both these clinical and histopathologic findings. 

The two most common types of granuloma annulare are generalized granuloma annulare (GGA) and localized granuloma annulare (LGA). The generalized form, as seen in our patient, is characterized by involvement of the trunk in addition to one or both the extremities. It is differentiated from the localized form, classical GA, by the wide distribution of lesions, protracted course with only rare spontaneous resolution, and poor response to therapy [5]. The duration of the skin eruption varies. In more than half of patients, it resolves spontaneously within two months to two years. However, cases of disseminated granuloma annulare may last three to four years, or as long as 10 years [7].

The etiology of GA is unknown, but the condition has been associated with multiple drugs including calcium channel blockers, angiotensin-converting enzyme inhibitors, lipid lowering agents, histamine H2 receptor antagonists, furosemide, carbamazepine, anti-tumor necrosis factor agents, and tricyclic antidepressants [7]. The only medication our patient had been taking that was associated with granulomatous dermatitis was amlodipine, but this is extremely rare. Furthermore, no changes had been made to our patient’s medication list for two years. Thus, it is likely that a delayed temporal relationship exists between the initiation of dupilumab and the development of GGA. A similar report was published documenting a five-year delay between starting ruxolitinib and developing GGA [8]. There have not yet been any documented cases of granulomatous dermatitis associated with dupilumab. 

Granulomatous dermatoses may be challenging to differentiate clinically but have unique histopathologic features [6]. The differential diagnosis includes interstitial granulomatous drug reaction (IGDR), but the lack of improvement four weeks after discontinuing dupilumab makes this less likely. Furthermore, histopathologic characteristics of IGDR such as vacuolar interface dermatitis, atypical lymphocytes, prominent eosinophils, a lack of neutrophils, and minimal collagen degeneration was not seen under the microscope in this patient [9]. The pharmaceutical company was contacted regarding this skin eruption and did not have any known documented cases of granulomatous dermatitis associated with dupilumab.

## Conclusions

Atopic dermatitis is a common and chronic condition that may require advanced therapies depending on its severity. Dupilumab is a novel monoclonal antibody that disconfigures the downstream potentiation of the Th2 pathway, the pathway involved in the pruritic component of the disease. Dupilumab is typically well tolerated by patients. However, as dupilumab is utilized more frequently for the treatment of atopic dermatitis, physicians must thoroughly document and report the potential side effects of this drug. 
